# Poly[tris{μ_2_-4-[4-(di­methyl­amino)­phenyl­diazenyl]benzene­sulfonato}tri­dioxane­tri­sodium(I)]

**DOI:** 10.1107/S1600536808008015

**Published:** 2008-03-29

**Authors:** Kazuyuki Sato, Hiroki Shibata, Jin Mizuguchi

**Affiliations:** a Department of Applied Physics, Graduate School of Engineering, Yokohama National University, 79-5 Tokiwadai, Hodogaya-ku, 240-8501 Yokohama, Japan

## Abstract

The title compound, [Na_3_(C_14_H_14_N_3_O_3_S)_3_(C_4_H_8_O_2_)_3_]_*n*_, is a polynuclear complex which includes, in the monomeric unit, three units of Na^I^-4′-dimethyl­amino­azobenzene-4-sulfonate [known as methyl orange (MO)] and three molecules of dioxane (C_4_H_8_O_2_). These constitute three kinds of Na^I^ centres, two of which are seven-coordinate while the third is five-coordinate. One of the seven-coordinate centres is coordinated by six O atoms from the sulfonate groups of four different MOs and by one O atom from dioxane. The other is coordinated by seven O atoms from the sulfonate groups of five different MOs. The five-coordinate centre is coordinated by three O atoms from the sulfonate groups of three different MOs and two O atoms from two different dioxanes. In the crystal structure, a one-dimensional polymer chain is formed along the *a* axis and this ensures the thermal stability of the title compound. It is also to be noted that the N=N bond lengths of the three azo groups are appreciably different [1.259 (4), 1.196 (4), and 1.253 (4) Å].

## Related literature

For general background on azo pigments, see: Herbst & Hunger (2004 ▶). For solvated methyl orange, see: Hanson (1973 ▶); Kennedy *et al.* (2004 ▶). For 4′-dimethyl­amino­azobenzene-4-sulfonic acid, see: Burke *et al.* (2004 ▶).
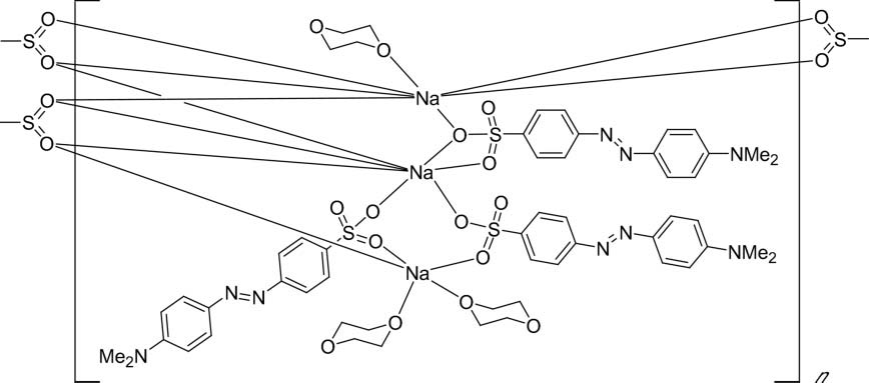


## Experimental

### 

#### Crystal data

[Na_3_(C_14_H_14_N_3_O_3_S)_3_(C_4_H_8_O_2_)_3_]
*M* *_r_* = 1246.34
Orthorhombic, 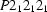

*a* = 8.4471 (6) Å
*b* = 15.5153 (10) Å
*c* = 44.488 (3) Å
*V* = 5830.6 (7) Å^3^
*Z* = 4
Cu *K*α radiation
μ = 2.01 mm^−1^
*T* = 93 (1) K
0.45 × 0.08 × 0.07 mm

#### Data collection

Rigaku R-AXIS RAPID diffractometer
Absorption correction: multi-scan (Higashi, 1995 ▶) *T*_min_ = 0.468, *T*_max_ = 0.869
47815 measured reflections
10385 independent reflections
6459 reflections with *F* ^2^ > 2σ(*F* ^2^)
*R*_int_ = 0.088

#### Refinement

*R*[*F* ^2^ > 2σ(*F* ^2^)] = 0.045
*wR*(*F* ^2^) = 0.085
*S* = 0.81
10385 reflections
764 parameters
H-atom parameters constrained
Δρ_max_ = 0.36 e Å^−3^
Δρ_min_ = −0.43 e Å^−3^
Absolute structure: Flack (1983 ▶), with 4457 Friedel pairs
Flack parameter: 0.006 (14)

### 

Data collection: *PROCESS-AUTO* (Rigaku, 1998 ▶); cell refinement: *PROCESS-AUTO*; data reduction: *CrystalStructure* (Rigaku/MSC & Rigaku, 2006 ▶); program(s) used to solve structure: *SIR2004* (Burla *et al.*, 2005 ▶); program(s) used to refine structure: *SHELXL97* (Sheldrick, 2008 ▶); molecular graphics: *ORTEPIII* (Burnett & Johnson, 1996 ▶); software used to prepare material for publication: *CrystalStructure*.

## Supplementary Material

Crystal structure: contains datablocks global, I. DOI: 10.1107/S1600536808008015/is2280sup1.cif

Structure factors: contains datablocks I. DOI: 10.1107/S1600536808008015/is2280Isup2.hkl

Additional supplementary materials:  crystallographic information; 3D view; checkCIF report

## Figures and Tables

**Table 1:** Selected bond lengths (Å)

Na1—O1	2.341 (2)
Na1—O4	2.394 (2)
Na1—O9^i^	2.258 (2)
Na1—O10	2.400 (2)
Na1—O12	2.280 (2)
Na2—O2	2.288 (2)
Na2—O5	2.371 (2)
Na2—O6^ii^	2.426 (2)
Na2—O7	2.459 (2)
Na2—O7^i^	2.643 (2)
Na2—O8	2.570 (2)
Na2—O9^i^	2.477 (2)
Na3—O2^i^	2.402 (2)
Na3—O3^i^	2.604 (2)
Na3—O4^ii^	2.446 (2)
Na3—O6^ii^	2.607 (2)
Na3—O7^i^	2.471 (2)
Na3—O8	2.449 (2)
Na3—O14	2.434 (2)

Symmetry codes: (i) 
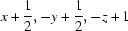
; (ii) 
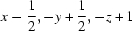
.

**Fig. 1.**
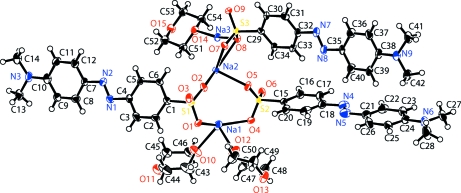
A view of the monomeric structure of (I), showing 50% displacement ellipsoids.

**Fig. 2.**
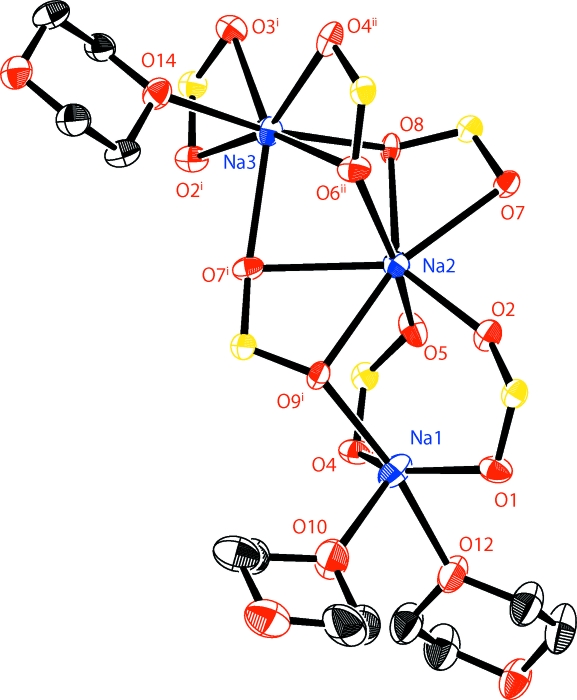
The three kinds of Na-complexes: five-coordinate Na1, seven-coordinate Na2 and Na3 [symmetry codes: (i) 1/2 + x, 1/2 - y, 1 - z; (ii) -1/2 + x, 1/2 - y, 1 - z].

**Fig. 3.**
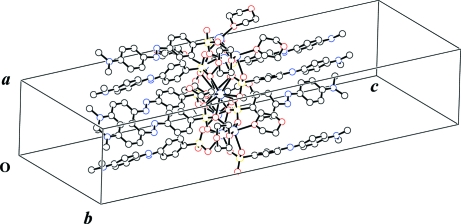
The crystal packing of the title compound.

## supplementary crystallographic information

### Comment

We are involved in the color generation mechanism of azo pigments typically characterized by the chromophore of the azo group (–N=N–). However, some types of azo pigments are also known to possess the hydrazone structure (=N—NH–), often leading to the formation of intramolecular hydrogen bonds (Herbst & Hunger, 2004). Methyl orange (MO) (NaO_3_SC_6_H_4_N=NC_6_H_4_NMe_2_), the skeleton of the title compound, is known as one of the classical azo pigments and its structure which includes solvent molecules as ligands have been determined by Hanson (1973) and Kennedy *et al.* (2004). These papers report the N/N distance to be about 1.24 Å, *i.e.* the typical distance of the –N=N– bond. On the other hand, the methyl orange derivative (HO_3_SC_6_H_4_N=NC_6_H_4_NMe_2_: 4'-dimethylaminoazobenzene-4-sulfonic acid) in which the Na atom is replace by H atom has been found to possess a zwitterionic structure in the solid state: ^-^O_3_SC_6_H_4_NH^+^=NC_6_H_4_NMe as characterized by a NH···O intermolecular hydrogen bond between the NH group of one molecule and one of the sulfate oxygen atoms (Burke *et al.*, 2004). This structure reveals a lengthening of the N=N bond to 1.307 (3) Å, indicating a hydrozone-like structure. In addition, the color of the crystal is no more orange but red violet. This motivated us to study the correlation between the crystal structure and the color in these two compounds. In the course of this study, a new MO complex has been found and its structure has been determined in the present investigation.

Figure 1 shows the *ORTEPIII* plot (Burnett & Johnson, 1996) of the monomeric unit of (I). The asymmetric unit includes three MO molecules together with three dioxane ones. These constitute three kinds of Na(I)-complexes, two of which are seven-coordinate and the other is five-coordinate. As shown in Fig. 2, there are two kinds of seven-coordinate complexes, one of which is chelated by six O atoms from the sulfonic group of four different MOs and also by one O atom from dioxane. The other is coordinated by seven O atoms from the sulfonic group of five different MOs. The five-coordinate complex includes three O atoms from the sulfonic group of three different MOs as well as two O atoms from the two different dioxanes. It is also important to note that the N/N bond lengths are typical of the azo group (–N=N–) but these are significantly different: 1.259 (4) Å for N1/N2, 1.196 (5) Å for N4/N5, and 1.253 (4) Å for N7/N8. The monomeric unit is extended alternately to form a one dimensional polymer along the *a* axis as shown in Fig. 3.

### Experimental

MO was purchased from Junsei Chemical Co., Ltd. Single crystals of (I) were grown by recrystallization from a dimethylacetamide solution by slow diffusion of 1,4-dioxane. After a week, a number of orange needle-like single crystals were obtained.

### Refinement

All H atoms were placed in geometrically idealized position and constrained to ride on their parent atoms, with C—H = 0.95, 0.98 and 0.99 Å, and *U*_iso_(H) = 1.2*U*_eq_(C).

### Crystal data

**Table:**

[Na_3_(C_14_H_14_N_3_O_3_S)_3_(C_4_H_8_O_2_)_3_]	*F*_000_ = 2616.00
*M**_r_* = 1246.34	*D*_x_ = 1.420 Mg m^−^^3^
Orthorhombic, *P*2_1_2_1_2_1_	Cu *K*α radiation λ = 1.54187 Å
Hall symbol: P 2ac 2ab	Cell parameters from 37788 reflections
*a* = 8.4471 (6) Å	θ = 3.0–69.5º
*b* = 15.5153 (10) Å	µ = 2.01 mm^−^^1^
*c* = 44.488 (3) Å	*T* = 93 (1) K
*V* = 5830.6 (7) Å^3^	Needle, orange
*Z* = 4	0.45 × 0.08 × 0.07 mm

### Data collection

**Table:**

Rigaku R-AXIS RAPID diffractometer	6459 reflections with *F*^2^ > 2σ(*F*^2^)
Detector resolution: 10.00 pixels mm^-1^	*R*_int_ = 0.088
ω scans	θ_max_ = 68.2º
Absorption correction: multi-scan(Higashi, 1995)	*h* = −9→9
*T*_min_ = 0.468, *T*_max_ = 0.869	*k* = −18→18
47815 measured reflections	*l* = −53→52
10385 independent reflections	

### Refinement

**Table:**

Refinement on *F*^2^	*w* = 1/[σ^2^(*F*_o_^2^) + (0.0379*P*)^2^] where *P* = (*F*_o_^2^ + 2*F*_c_^2^)/3
*R*[*F*^2^ > 2σ(*F*^2^)] = 0.045	(Δ/σ)_max_ = 0.001
*wR*(*F*^2^) = 0.085	Δρ_max_ = 0.36 e Å^−^^3^
*S* = 0.81	Δρ_min_ = −0.43 e Å^−^^3^
10385 reflections	Extinction correction: none
764 parameters	Absolute structure: Flack (1983), 4457 Friedel pairs
H-atom parameters constrained	Flack parameter: 0.006 (14)

### Special details

**Table:**

Geometry. All e.s.d.'s (except the e.s.d. in the dihedral angle between two l.s. planes) are estimated using the full covariance matrix. The cell e.s.d.'s are taken into account individually in the estimation of e.s.d.'s in distances, angles and torsion angles; correlations between e.s.d.'s in cell parameters are only used when they are defined by crystal symmetry. An approximate (isotropic) treatment of cell e.s.d.'s is used for estimating e.s.d.'s involving l.s. planes.
Refinement. Refinement of F^2^ against all reflections. The weighted R-factor wR and goodness of fit S are based on F^2^, conventional R-factors R are based on F, with F set to zero for negative F^2^. The threshold expression of F^2^ > σ(F^2^) is used only for calculating R-factors(gt) etc. and is not relevant to the choice of reflections for refinement. R-factors based on F^2^ are statistically about twice as large as those based on F, and R-factors based on all data will be even larger.

### Fractional atomic coordinates and isotropic or equivalent isotropic displacement parameters (Å^2^)

**Table:**

	*x*	*y*	*z*	*U*_iso_*/*U*_eq_	
S1	−0.22723 (12)	0.51838 (6)	0.500329 (18)	0.0235 (2)	
S2	0.24529 (12)	0.33481 (6)	0.444409 (17)	0.0228 (2)	
S3	−0.23478 (12)	0.15835 (6)	0.469265 (17)	0.0207 (2)	
Na1	0.14117 (17)	0.50896 (9)	0.48393 (3)	0.0364 (4)	
Na2	−0.05298 (15)	0.30286 (8)	0.49922 (3)	0.0222 (3)	
Na3	0.06100 (16)	0.11580 (8)	0.52800 (3)	0.0247 (3)	
O1	−0.1035 (2)	0.57736 (15)	0.49049 (5)	0.0289 (6)	
O2	−0.1910 (2)	0.42862 (14)	0.49340 (5)	0.0263 (6)	
O3	−0.3846 (2)	0.54162 (14)	0.48945 (5)	0.0272 (6)	
O4	0.3013 (2)	0.42185 (14)	0.45153 (5)	0.0288 (6)	
O5	0.0808 (2)	0.32228 (16)	0.45286 (5)	0.0307 (6)	
O6	0.3528 (3)	0.26919 (14)	0.45582 (4)	0.0284 (6)	
O7	−0.2901 (2)	0.24718 (13)	0.47370 (4)	0.0219 (6)	
O8	−0.0744 (2)	0.14658 (14)	0.48053 (4)	0.0212 (5)	
O9	−0.3498 (2)	0.09663 (14)	0.48117 (4)	0.0229 (6)	
O10	0.2588 (3)	0.61217 (16)	0.51733 (5)	0.0493 (8)	
O11	0.3014 (3)	0.7091 (2)	0.57093 (7)	0.0600 (9)	
O12	0.1835 (3)	0.60985 (16)	0.44754 (6)	0.0412 (7)	
O13	0.2640 (3)	0.69209 (16)	0.39299 (5)	0.0384 (7)	
O14	0.1498 (2)	0.07239 (16)	0.57779 (5)	0.0301 (6)	
O15	0.3011 (3)	0.03688 (16)	0.63339 (5)	0.0354 (7)	
N1	−0.2081 (3)	0.54629 (18)	0.63447 (6)	0.0288 (8)	
N2	−0.2712 (3)	0.48628 (18)	0.64929 (6)	0.0284 (7)	
N3	−0.2357 (4)	0.4979 (2)	0.77516 (6)	0.0410 (9)	
N4	0.2901 (4)	0.2991 (2)	0.30969 (7)	0.0450 (10)	
N5	0.2201 (4)	0.3531 (2)	0.29585 (8)	0.0466 (10)	
N6	0.2814 (4)	0.3307 (2)	0.16975 (6)	0.0425 (9)	
N7	−0.2721 (4)	0.12369 (19)	0.33631 (6)	0.0310 (8)	
N8	−0.1971 (3)	0.18020 (19)	0.32210 (6)	0.0335 (9)	
N9	−0.2472 (4)	0.1789 (2)	0.19640 (6)	0.0397 (9)	
C1	−0.2283 (4)	0.5248 (2)	0.54014 (7)	0.0235 (9)	
C2	−0.1781 (4)	0.5994 (2)	0.55415 (8)	0.0356 (11)	
C3	−0.1741 (4)	0.6044 (2)	0.58525 (7)	0.0401 (11)	
C4	−0.2232 (4)	0.5361 (2)	0.60262 (7)	0.0248 (9)	
C5	−0.2779 (4)	0.4619 (2)	0.58864 (7)	0.0266 (9)	
C6	−0.2802 (4)	0.4555 (2)	0.55754 (7)	0.0263 (9)	
C7	−0.2540 (5)	0.4925 (2)	0.68062 (7)	0.0283 (9)	
C8	−0.1563 (4)	0.5506 (2)	0.69568 (8)	0.0312 (10)	
C9	−0.1486 (4)	0.5526 (2)	0.72657 (8)	0.0363 (11)	
C10	−0.2412 (5)	0.4964 (2)	0.74404 (7)	0.0316 (10)	
C11	−0.3357 (4)	0.4355 (2)	0.72890 (8)	0.0366 (11)	
C12	−0.3399 (4)	0.4343 (2)	0.69779 (8)	0.0355 (10)	
C13	−0.1415 (4)	0.5631 (2)	0.79053 (7)	0.0426 (11)	
C14	−0.3432 (4)	0.4472 (2)	0.79285 (8)	0.0433 (11)	
C15	0.2514 (4)	0.3268 (2)	0.40466 (7)	0.0228 (8)	
C16	0.3362 (4)	0.2605 (2)	0.39107 (8)	0.0340 (10)	
C17	0.3430 (4)	0.2538 (2)	0.36013 (8)	0.0376 (11)	
C18	0.2644 (5)	0.3132 (2)	0.34274 (8)	0.0398 (11)	
C19	0.1751 (4)	0.3783 (2)	0.35570 (8)	0.0387 (11)	
C20	0.1685 (4)	0.3847 (2)	0.38701 (8)	0.0317 (10)	
C21	0.2468 (6)	0.3415 (2)	0.26331 (8)	0.0429 (12)	
C22	0.3391 (4)	0.2776 (2)	0.24986 (8)	0.0416 (12)	
C23	0.3525 (5)	0.2738 (2)	0.21889 (8)	0.0404 (11)	
C24	0.2711 (5)	0.3328 (2)	0.20068 (8)	0.0356 (10)	
C25	0.1777 (4)	0.3962 (2)	0.21499 (8)	0.0402 (12)	
C26	0.1683 (5)	0.3989 (2)	0.24557 (9)	0.0473 (12)	
C27	0.3730 (5)	0.2643 (2)	0.15458 (8)	0.0536 (13)	
C28	0.2072 (6)	0.3977 (2)	0.15193 (8)	0.0797 (18)	
C29	−0.2317 (4)	0.14013 (19)	0.42995 (7)	0.0183 (8)	
C30	−0.3588 (4)	0.0990 (2)	0.41650 (7)	0.0233 (9)	
C31	−0.3643 (4)	0.0927 (2)	0.38564 (7)	0.0282 (9)	
C32	−0.2470 (4)	0.1300 (2)	0.36811 (7)	0.0237 (9)	
C33	−0.1157 (4)	0.1677 (2)	0.38175 (7)	0.0276 (9)	
C34	−0.1070 (4)	0.1729 (2)	0.41273 (7)	0.0227 (9)	
C35	−0.2204 (4)	0.1761 (2)	0.29030 (7)	0.0283 (10)	
C36	−0.3160 (4)	0.1176 (2)	0.27532 (8)	0.0379 (11)	
C37	−0.3253 (4)	0.1181 (2)	0.24433 (8)	0.0389 (11)	
C38	−0.2368 (5)	0.1778 (2)	0.22709 (8)	0.0332 (10)	
C39	−0.1413 (4)	0.2362 (2)	0.24270 (8)	0.0339 (11)	
C40	−0.1333 (4)	0.2352 (2)	0.27349 (8)	0.0348 (10)	
C41	−0.3149 (5)	0.1046 (2)	0.18092 (7)	0.0518 (14)	
C42	−0.1464 (4)	0.2364 (2)	0.17905 (7)	0.0426 (11)	
C43	0.2370 (6)	0.7041 (2)	0.51826 (10)	0.0663 (15)	
C44	0.1934 (5)	0.7348 (2)	0.54849 (13)	0.0758 (17)	
C45	0.3257 (5)	0.6181 (2)	0.57015 (9)	0.0543 (14)	
C46	0.3743 (4)	0.5895 (2)	0.53932 (8)	0.0389 (11)	
C47	0.3421 (5)	0.6270 (3)	0.44005 (10)	0.0586 (14)	
C48	0.3665 (4)	0.6332 (3)	0.40808 (9)	0.0591 (14)	
C49	0.1042 (5)	0.6743 (3)	0.40154 (9)	0.0645 (15)	
C50	0.0770 (5)	0.6692 (2)	0.43346 (9)	0.0561 (13)	
C51	0.2248 (4)	0.1395 (2)	0.59486 (7)	0.0299 (9)	
C52	0.2227 (4)	0.1169 (2)	0.62798 (7)	0.0374 (10)	
C53	0.2281 (4)	−0.0283 (2)	0.61608 (7)	0.0345 (10)	
C54	0.2307 (4)	−0.0064 (2)	0.58293 (7)	0.0316 (10)	
H2	−0.1460	0.6475	0.5424	0.043*	
H3	−0.1371	0.6555	0.5947	0.048*	
H5	−0.3142	0.4149	0.6005	0.032*	
H6	−0.3168	0.4043	0.5481	0.032*	
H8	−0.0936	0.5898	0.6844	0.037*	
H9	−0.0798	0.5924	0.7362	0.044*	
H11	−0.3967	0.3952	0.7400	0.044*	
H12	−0.4035	0.3925	0.6879	0.043*	
H13a	−0.1810	0.6205	0.7853	0.051*	
H13b	−0.0306	0.5578	0.7843	0.051*	
H13c	−0.1496	0.5546	0.8123	0.051*	
H14a	−0.3174	0.3860	0.7906	0.052*	
H14b	−0.4519	0.4574	0.7860	0.052*	
H14c	−0.3338	0.4636	0.8141	0.052*	
H16	0.3900	0.2195	0.4032	0.041*	
H17	0.4015	0.2086	0.3510	0.045*	
H19	0.1191	0.4181	0.3434	0.046*	
H20	0.1071	0.4287	0.3962	0.038*	
H22	0.3928	0.2366	0.2620	0.050*	
H23	0.4173	0.2309	0.2099	0.048*	
H25	0.1213	0.4370	0.2033	0.048*	
H26	0.1049	0.4421	0.2548	0.057*	
H27a	0.4857	0.2726	0.1588	0.064*	
H27b	0.3551	0.2680	0.1329	0.064*	
H27c	0.3397	0.2074	0.1618	0.064*	
H28a	0.2426	0.3930	0.1310	0.096*	
H28b	0.2370	0.4543	0.1599	0.096*	
H28c	0.0920	0.3912	0.1528	0.096*	
H30	−0.4413	0.0753	0.4284	0.028*	
H31	−0.4490	0.0626	0.3763	0.034*	
H33	−0.0320	0.1899	0.3698	0.033*	
H34	−0.0177	0.1984	0.4222	0.027*	
H36	−0.3757	0.0768	0.2865	0.045*	
H37	−0.3920	0.0777	0.2344	0.047*	
H39	−0.0810	0.2773	0.2318	0.041*	
H40	−0.0672	0.2754	0.2835	0.042*	
H41a	−0.2576	0.0524	0.1868	0.062*	
H41b	−0.3059	0.1128	0.1592	0.062*	
H41c	−0.4268	0.0988	0.1864	0.062*	
H42a	−0.1809	0.2365	0.1580	0.051*	
H42b	−0.0365	0.2166	0.1802	0.051*	
H42c	−0.1540	0.2949	0.1872	0.051*	
H43a	0.1528	0.7205	0.5039	0.080*	
H43b	0.3361	0.7328	0.5119	0.080*	
H44a	0.1873	0.7985	0.5482	0.091*	
H44b	0.0871	0.7124	0.5536	0.091*	
H45a	0.2267	0.5883	0.5760	0.065*	
H45b	0.4089	0.6021	0.5848	0.065*	
H46a	0.4767	0.6166	0.5341	0.047*	
H46b	0.3893	0.5262	0.5393	0.047*	
H47a	0.4100	0.5806	0.4482	0.070*	
H47b	0.3749	0.6818	0.4496	0.070*	
H48a	0.4774	0.6508	0.4044	0.071*	
H48b	0.3519	0.5753	0.3992	0.071*	
H49a	0.0718	0.6190	0.3923	0.077*	
H49b	0.0352	0.7199	0.3931	0.077*	
H50a	0.0909	0.7270	0.4425	0.067*	
H50b	−0.0334	0.6506	0.4372	0.067*	
H51a	0.1684	0.1947	0.5916	0.036*	
H51b	0.3356	0.1468	0.5880	0.036*	
H52a	0.2757	0.1631	0.6395	0.045*	
H52b	0.1117	0.1129	0.6350	0.045*	
H53a	0.1171	−0.0356	0.6228	0.041*	
H53b	0.2840	−0.0836	0.6194	0.041*	
H54a	0.3417	−0.0013	0.5760	0.038*	
H54b	0.1793	−0.0532	0.5714	0.038*	

### Atomic displacement parameters (Å^2^)

**Table:**

	*U*^11^	*U*^22^	*U*^33^	*U*^12^	*U*^13^	*U*^23^
S1	0.0279 (6)	0.0264 (5)	0.0162 (4)	0.0019 (5)	−0.0006 (4)	0.0012 (3)
S2	0.0255 (6)	0.0286 (5)	0.0142 (4)	−0.0006 (5)	0.0006 (4)	0.0010 (3)
S3	0.0248 (6)	0.0238 (5)	0.0136 (4)	0.0007 (5)	0.0003 (4)	−0.0008 (3)
Na1	0.0469 (10)	0.0338 (8)	0.0285 (7)	0.0075 (8)	0.0062 (7)	0.0099 (6)
Na2	0.0234 (8)	0.0246 (7)	0.0187 (6)	−0.0009 (6)	0.0017 (6)	0.0002 (6)
Na3	0.0260 (9)	0.0277 (8)	0.0206 (7)	0.0023 (7)	0.0003 (6)	0.0011 (6)
O1	0.0337 (16)	0.0338 (15)	0.0193 (13)	−0.0084 (13)	0.0039 (12)	0.0015 (11)
O2	0.0323 (16)	0.0229 (13)	0.0239 (13)	0.0056 (12)	−0.0026 (11)	−0.0033 (10)
O3	0.0312 (16)	0.0347 (15)	0.0157 (12)	0.0060 (13)	−0.0040 (11)	−0.0007 (11)
O4	0.0364 (18)	0.0254 (14)	0.0244 (13)	−0.0094 (13)	0.0024 (12)	−0.0009 (10)
O5	0.0219 (15)	0.0525 (17)	0.0177 (13)	−0.0041 (14)	0.0037 (11)	−0.0025 (12)
O6	0.0396 (18)	0.0292 (14)	0.0165 (13)	0.0084 (14)	−0.0028 (12)	0.0020 (10)
O7	0.0292 (16)	0.0179 (12)	0.0186 (12)	0.0030 (12)	−0.0033 (11)	−0.0025 (9)
O8	0.0185 (14)	0.0325 (15)	0.0126 (12)	0.0041 (12)	−0.0056 (10)	0.0034 (10)
O9	0.0253 (15)	0.0228 (14)	0.0205 (13)	−0.0031 (12)	0.0044 (11)	0.0021 (10)
O10	0.065 (2)	0.0376 (17)	0.0452 (17)	0.0097 (18)	−0.0302 (17)	−0.0086 (13)
O11	0.057 (2)	0.058 (2)	0.065 (2)	−0.0100 (19)	0.0032 (19)	−0.0125 (17)
O12	0.0334 (19)	0.0430 (18)	0.0471 (17)	0.0066 (15)	0.0023 (14)	0.0237 (14)
O13	0.0374 (19)	0.0487 (17)	0.0292 (13)	0.0134 (17)	0.0061 (15)	0.0104 (12)
O14	0.0334 (17)	0.0327 (15)	0.0244 (14)	0.0021 (14)	−0.0016 (12)	0.0030 (12)
O15	0.046 (2)	0.0380 (16)	0.0221 (13)	−0.0015 (14)	−0.0059 (13)	0.0031 (12)
N1	0.034 (2)	0.0276 (19)	0.0251 (17)	0.0020 (16)	−0.0024 (15)	−0.0023 (14)
N2	0.027 (2)	0.0351 (19)	0.0231 (16)	0.0001 (18)	0.0032 (15)	0.0032 (14)
N3	0.044 (2)	0.057 (2)	0.0221 (17)	−0.008 (2)	0.0027 (19)	−0.0071 (16)
N4	0.040 (2)	0.043 (2)	0.053 (2)	−0.009 (2)	−0.014 (2)	0.0018 (17)
N5	0.042 (2)	0.039 (2)	0.059 (2)	−0.009 (2)	−0.016 (2)	0.0077 (18)
N6	0.071 (2)	0.042 (2)	0.0152 (16)	0.001 (2)	0.0008 (18)	0.0008 (15)
N7	0.035 (2)	0.035 (2)	0.0238 (17)	0.0062 (18)	0.0048 (17)	0.0006 (14)
N8	0.039 (2)	0.035 (2)	0.0261 (18)	0.0051 (18)	0.0060 (16)	−0.0020 (15)
N9	0.057 (2)	0.048 (2)	0.0141 (15)	−0.015 (2)	−0.0007 (19)	−0.0021 (14)
C1	0.026 (2)	0.021 (2)	0.0233 (19)	0.003 (2)	−0.0032 (18)	−0.0017 (15)
C2	0.059 (3)	0.024 (2)	0.024 (2)	−0.015 (2)	−0.003 (2)	0.0063 (17)
C3	0.066 (3)	0.032 (2)	0.022 (2)	−0.011 (2)	−0.003 (2)	−0.0080 (17)
C4	0.027 (2)	0.030 (2)	0.0169 (19)	0.002 (2)	−0.0005 (18)	−0.0023 (15)
C5	0.029 (2)	0.031 (2)	0.0201 (18)	−0.004 (2)	0.0019 (18)	0.0031 (15)
C6	0.029 (2)	0.028 (2)	0.0224 (19)	−0.0053 (19)	0.0001 (18)	−0.0075 (16)
C7	0.033 (2)	0.034 (2)	0.0183 (18)	−0.007 (2)	0.002 (2)	−0.0071 (16)
C8	0.037 (2)	0.031 (2)	0.025 (2)	−0.004 (2)	−0.0013 (19)	0.0040 (17)
C9	0.045 (3)	0.036 (2)	0.028 (2)	−0.009 (2)	−0.002 (2)	−0.0088 (18)
C10	0.033 (2)	0.040 (2)	0.022 (2)	0.006 (2)	−0.004 (2)	−0.0013 (17)
C11	0.038 (2)	0.048 (2)	0.023 (2)	−0.006 (2)	0.004 (2)	0.0031 (19)
C12	0.039 (2)	0.046 (2)	0.021 (2)	−0.005 (2)	−0.0018 (19)	−0.0050 (19)
C13	0.053 (3)	0.052 (2)	0.022 (2)	−0.003 (2)	−0.002 (2)	−0.004 (2)
C14	0.053 (3)	0.054 (2)	0.023 (2)	0.004 (2)	−0.004 (2)	0.004 (2)
C15	0.025 (2)	0.027 (2)	0.0160 (17)	−0.007 (2)	0.0012 (18)	−0.0028 (15)
C16	0.038 (2)	0.042 (2)	0.022 (2)	−0.013 (2)	−0.0032 (19)	−0.0002 (19)
C17	0.041 (2)	0.046 (2)	0.026 (2)	0.000 (2)	0.006 (2)	−0.005 (2)
C18	0.039 (3)	0.058 (3)	0.022 (2)	−0.025 (2)	0.008 (2)	−0.009 (2)
C19	0.035 (2)	0.052 (2)	0.029 (2)	−0.011 (2)	−0.014 (2)	0.017 (2)
C20	0.026 (2)	0.041 (2)	0.028 (2)	−0.001 (2)	−0.0018 (18)	0.0055 (19)
C21	0.054 (3)	0.052 (3)	0.023 (2)	−0.023 (3)	0.010 (2)	−0.007 (2)
C22	0.052 (3)	0.049 (2)	0.023 (2)	−0.020 (2)	−0.011 (2)	0.014 (2)
C23	0.051 (3)	0.043 (2)	0.027 (2)	−0.008 (2)	0.003 (2)	−0.0016 (19)
C24	0.049 (3)	0.036 (2)	0.0219 (19)	−0.012 (2)	−0.003 (2)	0.0025 (18)
C25	0.061 (3)	0.035 (2)	0.025 (2)	−0.005 (2)	0.001 (2)	0.0016 (18)
C26	0.066 (3)	0.044 (2)	0.032 (2)	−0.009 (2)	0.003 (2)	−0.009 (2)
C27	0.071 (3)	0.058 (3)	0.031 (2)	−0.014 (2)	0.006 (2)	−0.013 (2)
C28	0.163 (5)	0.053 (3)	0.022 (2)	0.017 (3)	0.004 (3)	0.004 (2)
C29	0.024 (2)	0.0144 (18)	0.0169 (17)	0.0065 (19)	−0.0037 (18)	0.0019 (13)
C30	0.026 (2)	0.026 (2)	0.0177 (19)	0.0006 (19)	0.0035 (17)	−0.0031 (16)
C31	0.025 (2)	0.031 (2)	0.028 (2)	−0.004 (2)	−0.0062 (18)	−0.0024 (18)
C32	0.029 (2)	0.026 (2)	0.0162 (18)	0.005 (2)	−0.0052 (19)	−0.0006 (15)
C33	0.025 (2)	0.036 (2)	0.022 (2)	0.003 (2)	0.0105 (17)	0.0057 (17)
C34	0.028 (2)	0.025 (2)	0.0153 (18)	0.0039 (19)	−0.0012 (16)	−0.0027 (16)
C35	0.033 (2)	0.040 (2)	0.0120 (18)	0.005 (2)	0.0011 (18)	−0.0019 (17)
C36	0.052 (3)	0.038 (2)	0.024 (2)	0.003 (2)	0.003 (2)	0.0045 (19)
C37	0.056 (3)	0.039 (2)	0.022 (2)	−0.001 (2)	−0.003 (2)	−0.0018 (19)
C38	0.041 (2)	0.040 (2)	0.0192 (19)	0.007 (2)	−0.001 (2)	−0.0003 (17)
C39	0.041 (3)	0.045 (2)	0.016 (2)	0.003 (2)	0.0072 (19)	0.0069 (18)
C40	0.035 (2)	0.043 (2)	0.027 (2)	0.000 (2)	−0.005 (2)	−0.0036 (19)
C41	0.084 (4)	0.057 (3)	0.014 (2)	−0.002 (2)	−0.002 (2)	0.000 (2)
C42	0.052 (3)	0.057 (2)	0.019 (2)	0.002 (2)	0.000 (2)	0.001 (2)
C43	0.078 (4)	0.028 (2)	0.092 (3)	0.000 (3)	−0.037 (3)	−0.009 (2)
C44	0.039 (3)	0.049 (3)	0.140 (5)	0.001 (2)	−0.012 (3)	−0.056 (3)
C45	0.066 (3)	0.047 (3)	0.050 (2)	−0.018 (2)	0.019 (2)	−0.014 (2)
C46	0.041 (3)	0.033 (2)	0.043 (2)	0.002 (2)	−0.008 (2)	−0.004 (2)
C47	0.032 (3)	0.079 (3)	0.065 (3)	0.001 (2)	0.001 (2)	0.039 (2)
C48	0.038 (3)	0.096 (4)	0.043 (2)	0.022 (3)	0.011 (2)	0.003 (2)
C49	0.044 (3)	0.117 (4)	0.033 (2)	0.046 (3)	0.005 (2)	0.010 (2)
C50	0.052 (3)	0.061 (3)	0.055 (3)	0.009 (2)	−0.002 (2)	0.025 (2)
C51	0.036 (2)	0.027 (2)	0.0266 (19)	−0.004 (2)	0.0048 (19)	0.0032 (15)
C52	0.051 (3)	0.038 (2)	0.0231 (19)	−0.005 (2)	−0.004 (2)	0.0004 (17)
C53	0.048 (3)	0.027 (2)	0.029 (2)	0.004 (2)	0.003 (2)	0.0061 (17)
C54	0.042 (2)	0.031 (2)	0.0223 (19)	−0.005 (2)	−0.001 (2)	−0.0013 (16)

### Geometric parameters (Å, °)

**Table:**

S1—O1	1.457 (2)	C14—H14b	0.980
S1—O2	1.459 (2)	C14—H14c	0.980
S1—O3	1.460 (2)	C15—C16	1.391 (5)
S1—C1	1.774 (3)	C15—C20	1.383 (5)
S2—O4	1.465 (2)	C16—C17	1.382 (5)
S2—O5	1.452 (2)	C16—H16	0.950
S2—O6	1.456 (2)	C17—C18	1.374 (5)
S2—C15	1.773 (3)	C17—H17	0.950
S3—O7	1.469 (2)	C18—C19	1.386 (5)
S3—O8	1.456 (2)	C19—C20	1.398 (5)
S3—O9	1.464 (2)	C19—H19	0.950
S3—C29	1.772 (3)	C20—H20	0.950
Na1—O1	2.341 (2)	C21—C22	1.396 (5)
Na1—O4	2.394 (2)	C21—C26	1.362 (5)
Na1—O9^i^	2.258 (2)	C22—C23	1.384 (5)
Na1—O10	2.400 (2)	C22—H22	0.950
Na1—O12	2.280 (2)	C23—C24	1.403 (5)
Na2—O2	2.288 (2)	C23—H23	0.950
Na2—O5	2.371 (2)	C24—C25	1.411 (5)
Na2—O6^ii^	2.426 (2)	C25—C26	1.363 (5)
Na2—O7	2.459 (2)	C25—H25	0.950
Na2—O7^i^	2.643 (2)	C26—H26	0.950
Na2—O8	2.570 (2)	C27—H27a	0.980
Na2—O9^i^	2.477 (2)	C27—H27b	0.980
Na3—O2^i^	2.402 (2)	C27—H27c	0.980
Na3—O3^i^	2.604 (2)	C28—H28a	0.980
Na3—O4^ii^	2.446 (2)	C28—H28b	0.980
Na3—O6^ii^	2.607 (2)	C28—H28c	0.980
Na3—O7^i^	2.471 (2)	C29—C30	1.385 (5)
Na3—O8	2.449 (2)	C29—C34	1.398 (4)
Na3—O14	2.434 (2)	C30—C31	1.377 (4)
O10—C43	1.439 (4)	C30—H30	0.950
O10—C46	1.425 (4)	C31—C32	1.387 (5)
O11—C44	1.410 (6)	C31—H31	0.950
O11—C45	1.427 (5)	C32—C33	1.393 (5)
O12—C47	1.406 (5)	C33—C34	1.383 (4)
O12—C50	1.432 (5)	C33—H33	0.950
O13—C48	1.426 (5)	C34—H34	0.950
O13—C49	1.430 (5)	C35—C36	1.385 (5)
O14—C51	1.437 (4)	C35—C40	1.393 (5)
O14—C54	1.420 (4)	C36—C37	1.381 (5)
O15—C52	1.428 (4)	C36—H36	0.950
O15—C53	1.413 (4)	C37—C38	1.416 (5)
N1—N2	1.259 (4)	C37—H37	0.950
N1—C4	1.431 (4)	C38—C39	1.398 (5)
N2—C7	1.405 (4)	C39—C40	1.372 (5)
N3—C10	1.385 (4)	C39—H39	0.950
N3—C13	1.457 (4)	C40—H40	0.950
N3—C14	1.436 (4)	C41—H41a	0.980
N4—N5	1.196 (4)	C41—H41b	0.980
N4—C18	1.502 (4)	C41—H41c	0.980
N5—C21	1.476 (5)	C42—H42a	0.980
N6—C24	1.379 (4)	C42—H42b	0.980
N6—C27	1.455 (5)	C42—H42c	0.980
N6—C28	1.450 (5)	C43—C44	1.473 (7)
N7—N8	1.253 (4)	C43—H43a	0.990
N7—C32	1.434 (4)	C43—H43b	0.990
N8—C35	1.430 (4)	C44—H44a	0.990
N9—C38	1.368 (4)	C44—H44b	0.990
N9—C41	1.460 (4)	C45—C46	1.499 (5)
N9—C42	1.455 (4)	C45—H45a	0.990
C1—C2	1.382 (4)	C45—H45b	0.990
C1—C6	1.395 (4)	C46—H46a	0.990
C2—C3	1.386 (4)	C46—H46b	0.990
C2—H2	0.950	C47—C48	1.440 (5)
C3—C4	1.376 (4)	C47—H47a	0.990
C3—H3	0.950	C47—H47b	0.990
C4—C5	1.388 (4)	C48—H48a	0.990
C5—C6	1.387 (4)	C48—H48b	0.990
C5—H5	0.950	C49—C50	1.441 (5)
C6—H6	0.950	C49—H49a	0.990
C7—C8	1.395 (5)	C49—H49b	0.990
C7—C12	1.387 (5)	C50—H50a	0.990
C8—C9	1.376 (5)	C50—H50b	0.990
C8—H8	0.950	C51—C52	1.515 (4)
C9—C10	1.406 (5)	C51—H51a	0.990
C9—H9	0.950	C51—H51b	0.990
C10—C11	1.408 (5)	C52—H52a	0.990
C11—C12	1.385 (5)	C52—H52b	0.990
C11—H11	0.950	C53—C54	1.514 (4)
C12—H12	0.950	C53—H53a	0.990
C13—H13a	0.980	C53—H53b	0.990
C13—H13b	0.980	C54—H54a	0.990
C13—H13c	0.980	C54—H54b	0.990
C14—H14a	0.980		
			
O3···H46a^iii^	2.581	H28a···H45b^xiv^	2.424
O3···H47a^iii^	2.596	H28b···N7^v^	2.650
O11···H49b^iv^	2.769	H40···H13a^viii^	2.671
O11···H50a^iv^	2.705	H41a···H19^vii^	2.742
O11···H50b^iv^	2.612	H41b···O13^vii^	2.649
O13···H3^iv^	2.567	H41b···H48b^vii^	2.687
O13···H41b^v^	2.649	H41c···H23^iii^	2.651
O13···H42a^v^	2.473	H42a···O13^vii^	2.473
O15···H13c^vi^	2.748	H42a···H49b^vii^	2.600
O15···H14c^vi^	2.615	H42c···H8^viii^	2.786
N7···H28b^vii^	2.650	H44a···H47b^x^	2.658
C40···H13a^viii^	2.784	H44a···H50b^iv^	2.572
C45···H28a^ix^	2.774	H44a···H54b^xv^	2.522
H2···H47b^x^	2.678	H44b···H47b^x^	2.434
H3···O13^x^	2.567	H45a···H28a^ix^	2.479
H8···H27a^ix^	2.584	H45b···H28a^ix^	2.424
H8···H42c^xi^	2.786	H46a···O3^xiii^	2.581
H13a···C40^xi^	2.784	H47a···O3^xiii^	2.596
H13a···H40^xi^	2.671	H47b···H2^iv^	2.678
H13c···O15^xii^	2.748	H47b···H44a^iv^	2.658
H13c···H52b^xii^	2.533	H47b···H44b^iv^	2.434
H14b···H26^xi^	2.456	H48b···H41b^v^	2.687
H14c···O15^xii^	2.615	H49b···O11^x^	2.769
H19···H41a^v^	2.742	H49b···H42a^v^	2.600
H23···H41c^xiii^	2.651	H50a···O11^x^	2.705
H26···H14b^viii^	2.456	H50b···O11^x^	2.612
H27a···H8^xiv^	2.584	H50b···H44a^x^	2.572
H28a···C45^xiv^	2.774	H52b···H13c^vi^	2.533
H28a···H45a^xiv^	2.479	H54b···H44a^xvi^	2.522
			
O1—S1—O2	112.68 (14)	S2—C15—C16	120.0 (2)
O1—S1—O3	113.50 (13)	S2—C15—C20	120.4 (2)
O1—S1—C1	105.59 (15)	C16—C15—C20	119.6 (3)
O2—S1—O3	110.90 (13)	C15—C16—C17	120.7 (3)
O2—S1—C1	105.39 (14)	C15—C16—H16	119.7
O3—S1—C1	108.21 (15)	C17—C16—H16	119.7
O4—S2—O5	112.08 (14)	C16—C17—C18	119.4 (3)
O4—S2—O6	111.58 (13)	C16—C17—H17	120.3
O4—S2—C15	105.72 (15)	C18—C17—H17	120.3
O5—S2—O6	114.39 (14)	N4—C18—C17	112.6 (3)
O5—S2—C15	106.05 (16)	N4—C18—C19	126.3 (3)
O6—S2—C15	106.29 (14)	C17—C18—C19	121.1 (3)
O7—S3—O8	111.56 (13)	C18—C19—C20	119.2 (3)
O7—S3—O9	110.74 (13)	C18—C19—H19	120.4
O7—S3—C29	106.68 (13)	C20—C19—H19	120.4
O8—S3—O9	114.29 (12)	C15—C20—C19	120.0 (3)
O8—S3—C29	107.83 (15)	C15—C20—H20	120.0
O9—S3—C29	105.22 (14)	C19—C20—H20	120.0
O1—Na1—O4	146.03 (9)	N5—C21—C22	126.3 (3)
O1—Na1—O9^i^	105.83 (9)	N5—C21—C26	114.5 (3)
O1—Na1—O10	89.19 (10)	C22—C21—C26	119.2 (3)
O1—Na1—O12	85.18 (9)	C21—C22—C23	120.2 (3)
O4—Na1—O9^i^	89.17 (8)	C21—C22—H22	119.9
O4—Na1—O10	121.03 (10)	C23—C22—H22	119.9
O4—Na1—O12	82.61 (9)	C22—C23—C24	120.5 (3)
O9^i^—Na1—O10	92.55 (9)	C22—C23—H23	119.8
O9^i^—Na1—O12	168.76 (11)	C24—C23—H23	119.8
O10—Na1—O12	85.21 (9)	N6—C24—C23	122.0 (3)
O2—Na2—O5	92.07 (9)	N6—C24—C25	120.1 (3)
O2—Na2—O6^ii^	108.62 (9)	C23—C24—C25	117.9 (3)
O2—Na2—O7	80.35 (8)	C24—C25—C26	120.3 (3)
O2—Na2—O7^i^	136.91 (8)	C24—C25—H25	119.9
O2—Na2—O8	137.09 (9)	C26—C25—H25	119.8
O2—Na2—O9^i^	81.71 (8)	C21—C26—C25	122.0 (3)
O5—Na2—O6^ii^	158.78 (9)	C21—C26—H26	119.0
O5—Na2—O7	91.80 (8)	C25—C26—H26	119.0
O5—Na2—O7^i^	91.91 (8)	N6—C27—H27a	109.5
O5—Na2—O8	82.64 (8)	N6—C27—H27b	109.5
O5—Na2—O9^i^	84.04 (8)	N6—C27—H27c	109.5
O6^ii^—Na2—O7	87.23 (8)	H27a—C27—H27b	109.5
O6^ii^—Na2—O7^i^	76.37 (8)	H27a—C27—H27c	109.5
O6^ii^—Na2—O8	78.98 (7)	H27b—C27—H27c	109.5
O6^ii^—Na2—O9^i^	103.11 (8)	N6—C28—H28a	109.5
O7—Na2—O7^i^	142.34 (8)	N6—C28—H28b	109.5
O7—Na2—O8	57.44 (7)	N6—C28—H28c	109.5
O7—Na2—O9^i^	161.42 (8)	H28a—C28—H28b	109.5
O7^i^—Na2—O8	85.95 (7)	H28a—C28—H28c	109.5
O7^i^—Na2—O9^i^	56.11 (7)	H28b—C28—H28c	109.5
O8—Na2—O9^i^	139.17 (8)	S3—C29—C30	119.3 (2)
O2^i^—Na3—O3^i^	57.24 (7)	S3—C29—C34	119.6 (2)
O2^i^—Na3—O4^ii^	149.46 (9)	C30—C29—C34	121.0 (2)
O2^i^—Na3—O6^ii^	153.40 (9)	C29—C30—C31	119.3 (3)
O2^i^—Na3—O7^i^	77.92 (8)	C29—C30—H30	120.3
O2^i^—Na3—O8	96.97 (8)	C31—C30—H30	120.4
O2^i^—Na3—O14	90.72 (8)	C30—C31—C32	120.5 (3)
O3^i^—Na3—O4^ii^	92.59 (8)	C30—C31—H31	119.8
O3^i^—Na3—O6^ii^	147.51 (9)	C32—C31—H31	119.8
O3^i^—Na3—O7^i^	135.08 (8)	N7—C32—C31	114.9 (3)
O3^i^—Na3—O8	90.47 (8)	N7—C32—C33	125.2 (3)
O3^i^—Na3—O14	87.56 (8)	C31—C32—C33	119.9 (2)
O4^ii^—Na3—O6^ii^	57.02 (7)	C32—C33—C34	120.1 (3)
O4^ii^—Na3—O7^i^	132.32 (8)	C32—C33—H33	120.0
O4^ii^—Na3—O8	87.07 (8)	C34—C33—H33	120.0
O4^ii^—Na3—O14	82.62 (8)	C29—C34—C33	119.0 (3)
O6^ii^—Na3—O7^i^	76.29 (8)	C29—C34—H34	120.5
O6^ii^—Na3—O8	77.86 (7)	C33—C34—H34	120.5
O6^ii^—Na3—O14	98.40 (8)	N8—C35—C36	125.9 (3)
O7^i^—Na3—O8	92.49 (7)	N8—C35—C40	115.4 (3)
O7^i^—Na3—O14	96.26 (8)	C36—C35—C40	118.7 (3)
O8—Na3—O14	169.39 (9)	C35—C36—C37	120.7 (3)
S1—O1—Na1	112.71 (13)	C35—C36—H36	119.7
S1—O2—Na2	153.80 (14)	C37—C36—H36	119.6
S1—O2—Na3^ii^	100.05 (11)	C36—C37—C38	120.9 (3)
Na2—O2—Na3^ii^	104.16 (9)	C36—C37—H37	119.6
S1—O3—Na3^ii^	91.59 (10)	C38—C37—H37	119.5
S2—O4—Na1	117.91 (12)	N9—C38—C37	121.0 (3)
S2—O4—Na3^i^	98.61 (11)	N9—C38—C39	121.6 (3)
Na1—O4—Na3^i^	114.66 (9)	C37—C38—C39	117.4 (3)
S2—O5—Na2	134.29 (14)	C38—C39—C40	121.1 (3)
S2—O6—Na2^i^	144.54 (12)	C38—C39—H39	119.5
S2—O6—Na3^i^	92.21 (11)	C40—C39—H39	119.4
Na2^i^—O6—Na3^i^	82.33 (7)	C35—C40—C39	121.2 (3)
S3—O7—Na2	97.62 (11)	C35—C40—H40	119.4
S3—O7—Na2^ii^	93.03 (10)	C39—C40—H40	119.4
S3—O7—Na3^ii^	164.82 (13)	N9—C41—H41a	109.5
Na2—O7—Na2^ii^	125.25 (8)	N9—C41—H41b	109.5
Na2—O7—Na3^ii^	97.27 (8)	N9—C41—H41c	109.5
Na2^ii^—O7—Na3^ii^	80.73 (7)	H41a—C41—H41b	109.5
S3—O8—Na2	93.35 (11)	H41a—C41—H41c	109.5
S3—O8—Na3	139.09 (12)	H41b—C41—H41c	109.5
Na2—O8—Na3	82.65 (7)	N9—C42—H42a	109.5
S3—O9—Na1^ii^	138.22 (14)	N9—C42—H42b	109.5
S3—O9—Na2^ii^	100.11 (11)	N9—C42—H42c	109.5
Na1^ii^—O9—Na2^ii^	101.03 (8)	H42a—C42—H42b	109.5
Na1—O10—C43	128.8 (2)	H42a—C42—H42c	109.5
Na1—O10—C46	122.9 (2)	H42b—C42—H42c	109.5
C43—O10—C46	108.2 (2)	O10—C43—C44	112.2 (3)
C44—O11—C45	110.8 (3)	O10—C43—H43a	109.2
Na1—O12—C47	116.6 (2)	O10—C43—H43b	109.2
Na1—O12—C50	130.8 (2)	C44—C43—H43a	109.2
C47—O12—C50	111.9 (3)	C44—C43—H43b	109.2
C48—O13—C49	108.9 (2)	H43a—C43—H43b	107.9
Na3—O14—C51	114.61 (18)	O11—C44—C43	113.2 (3)
Na3—O14—C54	122.24 (18)	O11—C44—H44a	108.9
C51—O14—C54	109.1 (2)	O11—C44—H44b	108.9
C52—O15—C53	109.2 (2)	C43—C44—H44a	108.9
N2—N1—C4	113.5 (2)	C43—C44—H44b	108.9
N1—N2—C7	115.2 (2)	H44a—C44—H44b	107.7
C10—N3—C13	119.9 (3)	O11—C45—C46	110.7 (3)
C10—N3—C14	121.2 (3)	O11—C45—H45a	109.5
C13—N3—C14	117.9 (2)	O11—C45—H45b	109.5
N5—N4—C18	109.3 (3)	C46—C45—H45a	109.5
N4—N5—C21	110.1 (3)	C46—C45—H45b	109.5
C24—N6—C27	120.9 (3)	H45a—C45—H45b	108.1
C24—N6—C28	120.1 (3)	O10—C46—C45	111.6 (3)
C27—N6—C28	119.0 (2)	O10—C46—H46a	109.3
N8—N7—C32	112.0 (2)	O10—C46—H46b	109.3
N7—N8—C35	113.5 (2)	C45—C46—H46a	109.3
C38—N9—C41	119.0 (3)	C45—C46—H46b	109.3
C38—N9—C42	120.0 (3)	H46a—C46—H46b	108.0
C41—N9—C42	117.6 (2)	O12—C47—C48	112.5 (3)
S1—C1—C2	119.7 (2)	O12—C47—H47a	109.1
S1—C1—C6	120.9 (2)	O12—C47—H47b	109.1
C2—C1—C6	119.4 (2)	C48—C47—H47a	109.1
C1—C2—C3	120.3 (3)	C48—C47—H47b	109.1
C1—C2—H2	119.8	H47a—C47—H47b	107.8
C3—C2—H2	119.9	O13—C48—C47	114.9 (3)
C2—C3—C4	120.7 (3)	O13—C48—H48a	108.6
C2—C3—H3	119.7	O13—C48—H48b	108.5
C4—C3—H3	119.6	C47—C48—H48a	108.5
N1—C4—C3	116.4 (2)	C47—C48—H48b	108.6
N1—C4—C5	124.4 (2)	H48a—C48—H48b	107.5
C3—C4—C5	119.2 (2)	O13—C49—C50	115.0 (3)
C4—C5—C6	120.7 (3)	O13—C49—H49a	108.5
C4—C5—H5	119.7	O13—C49—H49b	108.5
C6—C5—H5	119.7	C50—C49—H49a	108.5
C1—C6—C5	119.7 (3)	C50—C49—H49b	108.5
C1—C6—H6	120.2	H49a—C49—H49b	107.5
C5—C6—H6	120.2	O12—C50—C49	111.5 (3)
N2—C7—C8	125.6 (3)	O12—C50—H50a	109.3
N2—C7—C12	116.6 (3)	O12—C50—H50b	109.3
C8—C7—C12	117.8 (3)	C49—C50—H50a	109.3
C7—C8—C9	121.5 (3)	C49—C50—H50b	109.3
C7—C8—H8	119.3	H50a—C50—H50b	108.0
C9—C8—H8	119.3	O14—C51—C52	109.9 (2)
C8—C9—C10	120.8 (3)	O14—C51—H51a	109.7
C8—C9—H9	119.6	O14—C51—H51b	109.7
C10—C9—H9	119.6	C52—C51—H51a	109.7
N3—C10—C9	121.6 (3)	C52—C51—H51b	109.7
N3—C10—C11	120.5 (3)	H51a—C51—H51b	108.2
C9—C10—C11	117.8 (3)	O15—C52—C51	111.1 (2)
C10—C11—C12	120.1 (3)	O15—C52—H52a	109.4
C10—C11—H11	119.9	O15—C52—H52b	109.4
C12—C11—H11	119.9	C51—C52—H52a	109.4
C7—C12—C11	121.9 (3)	C51—C52—H52b	109.4
C7—C12—H12	119.1	H52a—C52—H52b	108.0
C11—C12—H12	119.1	O15—C53—C54	111.3 (2)
N3—C13—H13a	109.5	O15—C53—H53a	109.4
N3—C13—H13b	109.5	O15—C53—H53b	109.4
N3—C13—H13c	109.5	C54—C53—H53a	109.4
H13a—C13—H13b	109.5	C54—C53—H53b	109.3
H13a—C13—H13c	109.5	H53a—C53—H53b	108.0
H13b—C13—H13c	109.5	O14—C54—C53	110.1 (2)
N3—C14—H14a	109.5	O14—C54—H54a	109.6
N3—C14—H14b	109.5	O14—C54—H54b	109.7
N3—C14—H14c	109.5	C53—C54—H54a	109.6
H14a—C14—H14b	109.5	C53—C54—H54b	109.6
H14a—C14—H14c	109.5	H54a—C54—H54b	108.2
H14b—C14—H14c	109.5		
			
O1—S1—O2—Na2	69.6 (3)	O3^i^—Na3—O2^i^—Na2^i^	172.73 (12)
O1—S1—O2—Na3^ii^	−133.01 (12)	O2^i^—Na3—O4^ii^—S2^ii^	179.17 (15)
O2—S1—O1—Na1	−21.26 (17)	O2^i^—Na3—O4^ii^—Na1^ii^	−54.6 (2)
O1—S1—O3—Na3^ii^	132.15 (11)	O4^ii^—Na3—O2^i^—S1^i^	12.8 (2)
O3—S1—O1—Na1	−148.38 (12)	O4^ii^—Na3—O2^i^—Na2^i^	−177.25 (15)
O1—S1—C1—C2	27.1 (3)	O2^i^—Na3—O6^ii^—S2^ii^	−179.71 (17)
O1—S1—C1—C6	−153.2 (2)	O2^i^—Na3—O6^ii^—Na2	35.5 (2)
C1—S1—O1—Na1	93.26 (16)	O6^ii^—Na3—O2^i^—S1^i^	−159.98 (16)
O2—S1—O3—Na3^ii^	4.12 (13)	O6^ii^—Na3—O2^i^—Na2^i^	9.9 (2)
O3—S1—O2—Na2	−161.9 (2)	O2^i^—Na3—O7^i^—S3^i^	−164.7 (4)
O3—S1—O2—Na3^ii^	−4.53 (15)	O2^i^—Na3—O7^i^—Na2^i^	3.99 (8)
O2—S1—C1—C2	146.6 (2)	O2^i^—Na3—O7^i^—Na2	128.65 (7)
O2—S1—C1—C6	−33.8 (3)	O7^i^—Na3—O2^i^—S1^i^	−174.30 (12)
C1—S1—O2—Na2	−45.0 (3)	O7^i^—Na3—O2^i^—Na2^i^	−4.39 (8)
C1—S1—O2—Na3^ii^	112.34 (14)	O2^i^—Na3—O8—S3	162.35 (19)
O3—S1—C1—C2	−94.7 (3)	O2^i^—Na3—O8—Na2	−111.02 (7)
O3—S1—C1—C6	84.9 (3)	O8—Na3—O2^i^—S1^i^	−83.23 (12)
C1—S1—O3—Na3^ii^	−111.01 (13)	O8—Na3—O2^i^—Na2^i^	86.68 (10)
O4—S2—O5—Na2	−74.3 (2)	O2^i^—Na3—O14—C51	91.7 (2)
O5—S2—O4—Na1	13.40 (18)	O2^i^—Na3—O14—C54	−43.8 (2)
O5—S2—O4—Na3^i^	137.30 (11)	O14—Na3—O2^i^—S1^i^	89.45 (12)
O4—S2—O6—Na2^i^	72.9 (2)	O14—Na3—O2^i^—Na2^i^	−100.64 (10)
O4—S2—O6—Na3^i^	−6.98 (13)	O3^i^—Na3—O4^ii^—S2^ii^	−172.40 (11)
O6—S2—O4—Na1	−116.38 (14)	O3^i^—Na3—O4^ii^—Na1^ii^	−46.21 (11)
O6—S2—O4—Na3^i^	7.52 (14)	O4^ii^—Na3—O3^i^—S1^i^	−177.69 (11)
O4—S2—C15—C16	124.6 (3)	O3^i^—Na3—O6^ii^—S2^ii^	27.87 (19)
O4—S2—C15—C20	−57.2 (3)	O3^i^—Na3—O6^ii^—Na2	−116.94 (15)
C15—S2—O4—Na1	128.50 (16)	O6^ii^—Na3—O3^i^—S1^i^	162.96 (13)
C15—S2—O4—Na3^i^	−107.60 (15)	O3^i^—Na3—O7^i^—S3^i^	−168.2 (4)
O5—S2—O6—Na2^i^	−55.7 (2)	O3^i^—Na3—O7^i^—Na2^i^	0.56 (14)
O5—S2—O6—Na3^i^	−135.54 (12)	O3^i^—Na3—O7^i^—Na2	125.21 (11)
O6—S2—O5—Na2	54.0 (2)	O7^i^—Na3—O3^i^—S1^i^	1.22 (17)
O5—S2—C15—C16	−116.2 (3)	O3^i^—Na3—O8—S3	105.3 (2)
O5—S2—C15—C20	61.9 (3)	O3^i^—Na3—O8—Na2	−168.06 (7)
C15—S2—O5—Na2	170.82 (19)	O8—Na3—O3^i^—S1^i^	95.22 (11)
O6—S2—C15—C16	5.9 (3)	O3^i^—Na3—O14—C51	148.9 (2)
O6—S2—C15—C20	−175.9 (2)	O3^i^—Na3—O14—C54	13.3 (2)
C15—S2—O6—Na2^i^	−172.4 (2)	O14—Na3—O3^i^—S1^i^	−95.20 (11)
C15—S2—O6—Na3^i^	107.79 (14)	O4^ii^—Na3—O6^ii^—S2^ii^	4.63 (8)
O7—S3—O8—Na2	1.51 (12)	O4^ii^—Na3—O6^ii^—Na2	−140.18 (10)
O7—S3—O8—Na3	84.2 (2)	O6^ii^—Na3—O4^ii^—S2^ii^	−4.65 (8)
O8—S3—O7—Na2	−1.59 (12)	O6^ii^—Na3—O4^ii^—Na1^ii^	121.54 (12)
O8—S3—O7—Na2^ii^	−127.76 (10)	O4^ii^—Na3—O7^i^—S3^i^	10.4 (5)
O8—S3—O7—Na3^ii^	167.1 (4)	O4^ii^—Na3—O7^i^—Na2^i^	179.09 (10)
O7—S3—O9—Na1^ii^	−120.22 (18)	O4^ii^—Na3—O7^i^—Na2	−56.26 (11)
O7—S3—O9—Na2^ii^	−0.81 (13)	O7^i^—Na3—O4^ii^—S2^ii^	8.63 (17)
O9—S3—O7—Na2	126.91 (10)	O7^i^—Na3—O4^ii^—Na1^ii^	134.83 (10)
O9—S3—O7—Na2^ii^	0.75 (12)	O4^ii^—Na3—O8—S3	12.7 (2)
O9—S3—O7—Na3^ii^	−64.4 (5)	O4^ii^—Na3—O8—Na2	99.38 (7)
O7—S3—C29—C30	−97.1 (2)	O8—Na3—O4^ii^—S2^ii^	−82.07 (11)
O7—S3—C29—C34	78.2 (2)	O8—Na3—O4^ii^—Na1^ii^	44.13 (10)
C29—S3—O7—Na2	−119.11 (14)	O4^ii^—Na3—O14—C51	−118.2 (2)
C29—S3—O7—Na2^ii^	114.73 (14)	O4^ii^—Na3—O14—C54	106.2 (2)
C29—S3—O7—Na3^ii^	49.6 (5)	O14—Na3—O4^ii^—S2^ii^	100.40 (12)
O8—S3—O9—Na1^ii^	6.8 (2)	O14—Na3—O4^ii^—Na1^ii^	−133.41 (11)
O8—S3—O9—Na2^ii^	126.21 (11)	O6^ii^—Na3—O7^i^—S3^i^	21.8 (4)
O9—S3—O8—Na2	−125.08 (11)	O6^ii^—Na3—O7^i^—Na2^i^	−169.47 (8)
O9—S3—O8—Na3	−42.4 (2)	O6^ii^—Na3—O7^i^—Na2	−44.81 (6)
O8—S3—C29—C30	142.9 (2)	O7^i^—Na3—O6^ii^—S2^ii^	−165.30 (10)
O8—S3—C29—C34	−41.7 (2)	O7^i^—Na3—O6^ii^—Na2	49.89 (7)
C29—S3—O8—Na2	118.33 (12)	O6^ii^—Na3—O8—S3	−44.1 (2)
C29—S3—O8—Na3	−159.02 (18)	O6^ii^—Na3—O8—Na2	42.50 (7)
O9—S3—C29—C30	20.5 (3)	O8—Na3—O6^ii^—S2^ii^	99.06 (10)
O9—S3—C29—C34	−164.1 (2)	O8—Na3—O6^ii^—Na2	−45.75 (7)
C29—S3—O9—Na1^ii^	124.88 (19)	O6^ii^—Na3—O14—C51	−63.2 (2)
C29—S3—O9—Na2^ii^	−115.70 (13)	O6^ii^—Na3—O14—C54	161.2 (2)
O1—Na1—O4—S2	−62.5 (2)	O14—Na3—O6^ii^—S2^ii^	−70.85 (11)
O1—Na1—O4—Na3^i^	−177.90 (14)	O14—Na3—O6^ii^—Na2	144.33 (8)
O4—Na1—O1—S1	80.1 (2)	O7^i^—Na3—O8—S3	−119.5 (2)
O1—Na1—O9^i^—S3^i^	−171.62 (17)	O7^i^—Na3—O8—Na2	−32.89 (7)
O1—Na1—O9^i^—Na2	69.27 (10)	O8—Na3—O7^i^—S3^i^	98.7 (4)
O9^i^—Na1—O1—S1	−33.09 (15)	O8—Na3—O7^i^—Na2^i^	−92.61 (8)
O1—Na1—O10—C43	−44.4 (3)	O8—Na3—O7^i^—Na2	32.05 (7)
O1—Na1—O10—C46	139.5 (2)	O7^i^—Na3—O14—C51	13.8 (2)
O10—Na1—O1—S1	−125.51 (13)	O7^i^—Na3—O14—C54	−121.8 (2)
O1—Na1—O12—C47	157.4 (2)	O14—Na3—O7^i^—S3^i^	−75.4 (5)
O1—Na1—O12—C50	−12.4 (3)	O14—Na3—O7^i^—Na2^i^	93.38 (8)
O12—Na1—O1—S1	149.23 (14)	O14—Na3—O7^i^—Na2	−141.96 (8)
O4—Na1—O9^i^—S3^i^	39.3 (2)	O8—Na3—O14—C51	−131.7 (5)
O4—Na1—O9^i^—Na2	−79.82 (9)	O8—Na3—O14—C54	92.8 (5)
O9^i^—Na1—O4—S2	55.36 (14)	O14—Na3—O8—S3	26.1 (6)
O9^i^—Na1—O4—Na3^i^	−60.09 (11)	O14—Na3—O8—Na2	112.8 (5)
O4—Na1—O10—C43	119.3 (3)	Na1—O10—C43—C44	127.6 (3)
O4—Na1—O10—C46	−56.9 (2)	Na1—O10—C46—C45	−125.2 (2)
O10—Na1—O4—S2	147.83 (13)	C43—O10—C46—C45	57.9 (4)
O10—Na1—O4—Na3^i^	32.39 (14)	C46—O10—C43—C44	−55.8 (4)
O4—Na1—O12—C47	−54.4 (2)	C44—O11—C45—C46	54.0 (4)
O4—Na1—O12—C50	135.8 (3)	C45—O11—C44—C43	−53.0 (4)
O12—Na1—O4—S2	−132.32 (15)	Na1—O12—C47—C48	136.3 (3)
O12—Na1—O4—Na3^i^	112.24 (11)	Na1—O12—C50—C49	−138.0 (3)
O9^i^—Na1—O10—C43	−150.2 (3)	C47—O12—C50—C49	51.9 (4)
O9^i^—Na1—O10—C46	33.7 (2)	C50—O12—C47—C48	−52.0 (4)
O10—Na1—O9^i^—S3^i^	−81.7 (2)	C48—O13—C49—C50	51.0 (4)
O10—Na1—O9^i^—Na2	159.15 (9)	C49—O13—C48—C47	−50.5 (4)
O9^i^—Na1—O12—C47	−11.1 (6)	Na3—O14—C51—C52	160.1 (2)
O9^i^—Na1—O12—C50	179.1 (4)	Na3—O14—C54—C53	−163.4 (2)
O12—Na1—O9^i^—S3^i^	−3.6 (6)	C51—O14—C54—C53	58.9 (3)
O12—Na1—O9^i^—Na2	−122.7 (5)	C54—O14—C51—C52	−58.7 (3)
O10—Na1—O12—C47	67.8 (2)	C52—O15—C53—C54	57.6 (3)
O10—Na1—O12—C50	−102.0 (3)	C53—O15—C52—C51	−57.2 (3)
O12—Na1—O10—C43	40.9 (3)	N2—N1—C4—C3	172.3 (3)
O12—Na1—O10—C46	−135.3 (2)	N2—N1—C4—C5	−10.1 (5)
O2—Na2—O5—S2	116.5 (2)	C4—N1—N2—C7	177.9 (3)
O5—Na2—O2—S1	−107.2 (3)	N1—N2—C7—C8	−9.9 (5)
O5—Na2—O2—Na3^ii^	95.84 (10)	N1—N2—C7—C12	171.5 (3)
O2—Na2—O6^ii^—S2^ii^	96.0 (2)	C13—N3—C10—C9	4.8 (5)
O2—Na2—O6^ii^—Na3	179.03 (8)	C13—N3—C10—C11	−178.0 (3)
O6^ii^—Na2—O2—S1	77.6 (3)	C14—N3—C10—C9	173.0 (3)
O6^ii^—Na2—O2—Na3^ii^	−79.37 (10)	C14—N3—C10—C11	−9.8 (5)
O2—Na2—O7—S3	172.89 (11)	N5—N4—C18—C17	−179.3 (3)
O2—Na2—O7—Na2^ii^	−87.92 (11)	N5—N4—C18—C19	−0.3 (5)
O2—Na2—O7—Na3^ii^	−4.16 (8)	C18—N4—N5—C21	178.7 (3)
O7—Na2—O2—S1	161.4 (3)	N4—N5—C21—C22	0.1 (5)
O7—Na2—O2—Na3^ii^	4.37 (8)	N4—N5—C21—C26	178.3 (3)
O2—Na2—O7^i^—S3^i^	−14.05 (16)	C27—N6—C24—C23	2.5 (6)
O2—Na2—O7^i^—Na2^i^	−115.59 (13)	C27—N6—C24—C25	−178.0 (3)
O2—Na2—O7^i^—Na3	152.02 (12)	C28—N6—C24—C23	−174.3 (3)
O7^i^—Na2—O2—S1	−12.2 (4)	C28—N6—C24—C25	5.2 (5)
O7^i^—Na2—O2—Na3^ii^	−169.15 (9)	N8—N7—C32—C31	157.9 (3)
O2—Na2—O8—S3	−12.78 (17)	N8—N7—C32—C33	−23.8 (4)
O2—Na2—O8—Na3	−151.88 (12)	C32—N7—N8—C35	−179.6 (3)
O8—Na2—O2—S1	171.4 (2)	N7—N8—C35—C36	0.6 (5)
O8—Na2—O2—Na3^ii^	14.43 (16)	N7—N8—C35—C40	−177.1 (3)
O2—Na2—O9^i^—S3^i^	171.19 (11)	C41—N9—C38—C37	−16.5 (5)
O2—Na2—O9^i^—Na1	−45.06 (9)	C41—N9—C38—C39	164.7 (3)
O9^i^—Na2—O2—S1	−23.5 (3)	C42—N9—C38—C37	−175.4 (3)
O9^i^—Na2—O2—Na3^ii^	179.51 (9)	C42—N9—C38—C39	5.8 (5)
O5—Na2—O6^ii^—S2^ii^	−70.6 (4)	S1—C1—C2—C3	−178.2 (2)
O5—Na2—O6^ii^—Na3	12.4 (2)	S1—C1—C6—C5	179.2 (2)
O6^ii^—Na2—O5—S2	−76.2 (3)	C2—C1—C6—C5	−1.1 (5)
O5—Na2—O7—S3	81.09 (10)	C6—C1—C2—C3	2.2 (5)
O5—Na2—O7—Na2^ii^	−179.71 (11)	C1—C2—C3—C4	−1.4 (5)
O5—Na2—O7—Na3^ii^	−95.95 (8)	C2—C3—C4—N1	177.4 (3)
O7—Na2—O5—S2	−163.1 (2)	C2—C3—C4—C5	−0.3 (5)
O5—Na2—O7^i^—S3^i^	81.02 (10)	N1—C4—C5—C6	−176.2 (3)
O5—Na2—O7^i^—Na2^i^	−20.52 (12)	C3—C4—C5—C6	1.4 (5)
O5—Na2—O7^i^—Na3	−112.91 (8)	C4—C5—C6—C1	−0.6 (5)
O7^i^—Na2—O5—S2	−20.6 (2)	N2—C7—C8—C9	179.2 (3)
O5—Na2—O8—S3	−97.88 (10)	N2—C7—C12—C11	−178.4 (3)
O5—Na2—O8—Na3	123.02 (8)	C8—C7—C12—C11	2.8 (5)
O8—Na2—O5—S2	−106.3 (2)	C12—C7—C8—C9	−2.1 (5)
O5—Na2—O9^i^—S3^i^	−95.83 (11)	C7—C8—C9—C10	−0.9 (5)
O5—Na2—O9^i^—Na1	47.92 (9)	C8—C9—C10—N3	−179.6 (3)
O9^i^—Na2—O5—S2	35.0 (2)	C8—C9—C10—C11	3.1 (5)
O6^ii^—Na2—O7—S3	−77.69 (10)	N3—C10—C11—C12	−179.7 (3)
O6^ii^—Na2—O7—Na2^ii^	21.50 (11)	C9—C10—C11—C12	−2.4 (5)
O6^ii^—Na2—O7—Na3^ii^	105.26 (8)	C10—C11—C12—C7	−0.6 (5)
O7—Na2—O6^ii^—S2^ii^	17.2 (2)	S2—C15—C16—C17	−179.5 (2)
O7—Na2—O6^ii^—Na3	100.18 (7)	S2—C15—C20—C19	179.4 (2)
O6^ii^—Na2—O7^i^—S3^i^	−116.86 (10)	C16—C15—C20—C19	−2.4 (5)
O6^ii^—Na2—O7^i^—Na2^i^	141.60 (11)	C20—C15—C16—C17	2.3 (5)
O6^ii^—Na2—O7^i^—Na3	49.21 (7)	C15—C16—C17—C18	−0.3 (5)
O7^i^—Na2—O6^ii^—S2^ii^	−128.6 (2)	C16—C17—C18—N4	177.4 (3)
O7^i^—Na2—O6^ii^—Na3	−45.63 (6)	C16—C17—C18—C19	−1.7 (6)
O6^ii^—Na2—O8—S3	92.78 (10)	N4—C18—C19—C20	−177.3 (3)
O6^ii^—Na2—O8—Na3	−46.31 (8)	C17—C18—C19—C20	1.6 (6)
O8—Na2—O6^ii^—S2^ii^	−40.2 (2)	C18—C19—C20—C15	0.5 (5)
O8—Na2—O6^ii^—Na3	42.83 (6)	N5—C21—C22—C23	179.3 (4)
O6^ii^—Na2—O9^i^—S3^i^	63.89 (11)	N5—C21—C26—C25	−178.8 (3)
O6^ii^—Na2—O9^i^—Na1	−152.36 (9)	C22—C21—C26—C25	−0.4 (6)
O9^i^—Na2—O6^ii^—S2^ii^	−178.4 (2)	C26—C21—C22—C23	1.2 (6)
O9^i^—Na2—O6^ii^—Na3	−95.42 (8)	C21—C22—C23—C24	−1.3 (6)
O7—Na2—O7^i^—S3^i^	176.44 (11)	C22—C23—C24—N6	−179.8 (3)
O7—Na2—O7^i^—Na2^i^	74.90 (17)	C22—C23—C24—C25	0.7 (6)
O7—Na2—O7^i^—Na3	−17.49 (14)	N6—C24—C25—C26	−179.5 (3)
O7^i^—Na2—O7—S3	−14.36 (18)	C23—C24—C25—C26	−0.0 (5)
O7^i^—Na2—O7—Na2^ii^	84.84 (16)	C24—C25—C26—C21	−0.1 (5)
O7^i^—Na2—O7—Na3^ii^	168.60 (11)	S3—C29—C30—C31	173.3 (2)
O7—Na2—O8—S3	−1.00 (8)	S3—C29—C34—C33	−172.0 (2)
O7—Na2—O8—Na3	−140.09 (9)	C30—C29—C34—C33	3.3 (4)
O8—Na2—O7—S3	1.00 (8)	C34—C29—C30—C31	−2.0 (5)
O8—Na2—O7—Na2^ii^	100.19 (11)	C29—C30—C31—C32	−2.5 (5)
O8—Na2—O7—Na3^ii^	−176.05 (9)	C30—C31—C32—N7	−176.1 (3)
O7—Na2—O9^i^—S3^i^	−173.6 (2)	C30—C31—C32—C33	5.5 (5)
O7—Na2—O9^i^—Na1	−29.9 (3)	N7—C32—C33—C34	177.6 (3)
O9^i^—Na2—O7—S3	157.6 (2)	C31—C32—C33—C34	−4.2 (5)
O9^i^—Na2—O7—Na2^ii^	−103.2 (2)	C32—C33—C34—C29	−0.2 (4)
O9^i^—Na2—O7—Na3^ii^	−19.4 (2)	N8—C35—C36—C37	−177.9 (3)
O7^i^—Na2—O8—S3	169.67 (9)	N8—C35—C40—C39	178.0 (3)
O7^i^—Na2—O8—Na3	30.57 (7)	C36—C35—C40—C39	0.2 (4)
O8—Na2—O7^i^—S3^i^	163.50 (9)	C40—C35—C36—C37	−0.3 (5)
O8—Na2—O7^i^—Na2^i^	61.96 (11)	C35—C36—C37—C38	0.4 (6)
O8—Na2—O7^i^—Na3	−30.42 (6)	C36—C37—C38—N9	−179.3 (3)
O7^i^—Na2—O9^i^—S3^i^	0.50 (8)	C36—C37—C38—C39	−0.5 (5)
O7^i^—Na2—O9^i^—Na1	144.25 (10)	N9—C38—C39—C40	179.2 (3)
O9^i^—Na2—O7^i^—S3^i^	−0.50 (8)	C37—C38—C39—C40	0.4 (5)
O9^i^—Na2—O7^i^—Na2^i^	−102.04 (12)	C38—C39—C40—C35	−0.2 (5)
O9^i^—Na2—O7^i^—Na3	165.58 (9)	O10—C43—C44—O11	54.7 (5)
O8—Na2—O9^i^—S3^i^	−24.36 (16)	O11—C45—C46—O10	−58.4 (4)
O8—Na2—O9^i^—Na1	119.39 (12)	O12—C47—C48—O13	52.9 (5)
O9^i^—Na2—O8—S3	−169.85 (11)	O13—C49—C50—O12	−52.9 (5)
O9^i^—Na2—O8—Na3	51.05 (13)	O14—C51—C52—O15	58.4 (3)
O2^i^—Na3—O3^i^—S1^i^	−2.78 (9)	O15—C53—C54—O14	−59.5 (4)
O3^i^—Na3—O2^i^—S1^i^	2.82 (9)		

Symmetry codes: (i) *x*+1/2, −*y*+1/2, −*z*+1; (ii) *x*−1/2, −*y*+1/2, −*z*+1; (iii) *x*−1, *y*, *z*; (iv) *x*+1/2, −*y*+3/2, −*z*+1; (v) −*x*, *y*+1/2, −*z*+1/2; (vi) −*x*, *y*−1/2, −*z*+3/2; (vii) −*x*, *y*−1/2, −*z*+1/2; (viii) −*x*−1/2, −*y*+1, *z*−1/2; (ix) −*x*+1/2, −*y*+1, *z*+1/2; (x) *x*−1/2, −*y*+3/2, −*z*+1; (xi) −*x*−1/2, −*y*+1, *z*+1/2; (xii) −*x*, *y*+1/2, −*z*+3/2; (xiii) *x*+1, *y*, *z*; (xiv) −*x*+1/2, −*y*+1, *z*−1/2; (xv) *x*, *y*+1, *z*; (xvi) *x*, *y*−1, *z*.
